# Single-cell and bulk sequencing analyses reveal the immune suppressive role of PTPN6 in glioblastoma

**DOI:** 10.18632/aging.205052

**Published:** 2023-09-21

**Authors:** Xiaonan Zhang, Jie Chen, Ming Zhang, Saisai Liu, Tao Wang, Tianyu Wu, Baiqing Li, Shidi Zhao, Hongtao Wang, Li Li, Chun Wang, Li Huang

**Affiliations:** 1 Department of Pathophysiology, Bengbu Medical College, Longzihu, Bengbu 233030, Anhui, P.R. China; 2 Bengbu Medical College Key Laboratory of Cardiovascular and Cerebrovascular Diseases, Longzihu, Bengbu 233030, Anhui, P.R. China; 3 Research Laboratory Centre, Guizhou Provincial People’s Hospital, Guizhou University, Nanming, Guiyang 550025, Guizhou, P.R. China; 4 Anhui Province Key Laboratory of Immunology in Chronic Diseases, Bengbu Medical College, Longzihu, Bengbu 233030, Anhui, P.R. China; 5 Department of General Practice, The Second Affiliated Hospital of Bengbu Medical College, Huaishang, Bengbu 233040, Anhui, P.R. China; 6 Department of Endocrinology, The Second Affiliated Hospital of Bengbu Medical College, Huaishang, Bengbu 233040, Anhui, P.R. China

**Keywords:** immunotherapy, GBM

## Abstract

Glioblastoma (GBM) is a highly malignant brain cancer with a poor prognosis despite standard treatments. This investigation aimed to explore the feasibility of PTPN6 to combat GBM with immunotherapy. Our study employed a comprehensive analysis of publicly available datasets and functional experiments to assess PTPN6 gene expression, prognostic value, and related immune characteristics in glioma. We evaluated the influence of PTPN6 expression on CD8+ T cell exhaustion, immune suppression, and tumor growth in human GBM samples and mouse models. Our findings demonstrated that PTPN6 overexpression played an oncogenic role in GBM and was associated with advanced tumor grades and unfavorable clinical outcomes. In human GBM samples, PTPN6 upregulation showed a strong association with immunosuppressive formation and CD8+ T cell dysfunction, whereas, in mice, it hindered CD8+ T cell infiltration. Moreover, PTPN6 facilitated cell cycle progression, inhibited apoptosis, and promoted glioma cell proliferation, tumor growth, and colony formation in mice. The outcomes of our study indicate that PTPN6 is a promising immunotherapeutic target for the treatment of GBM. Inhibition of PTPN6 could enhance CD8+ T cell infiltration and improve antitumor immune response, thus leading to better clinical outcomes for GBM patients.

## INTRODUCTION

GBM is an unconquerable brain carcinoma commonly managed through surgical resection, chemotherapy, radiotherapy, and targeted therapy [1, 2]. Although notable advancements in diacrisis and treatment, GBM carries a grim prognosis with an overall survival rate of approximately 12-15 months post-diagnosis and a five-year survival rate of less than 10% [3]. While immunotherapy, such as immune checkpoint inhibitors (ICIs), has shown promise as an additional treatment option for several cancer types by altering the tumor microenvironment (TME) [4–9], only 10% of GBM patients benefit from this approach [10]. Therefore, identifying novel therapeutic targets for GBM is an urgent and necessary task.

PTPN6 involves various processes, including cell differentiation, growth, and oncogenic transformation [11]. It also plays a role in antigen cross-presentation for immune evasion [12] and is critical for ligand-mediated CD22 regulation in BCR-ligated B cells [13]. Previous research has shown that PTPN6 may promote chemosensitivity in colorectal cancer cells by inhibiting the SP1/MAPK signaling pathway (14) and enhancing macrophage effector function to bolster antitumor immunity [14]. However, the specific mechanisms of PTPN6 in GBM are still unknown. Here, we systematically investigated the functions of PTPN6 towards immune response in GBM and indicated that PTPN6 might be leveraged as a promising new therapeutic target for GBM treatment.

## RESULTS

### PTPN6 is overexpressed and identified as a prognostic marker in GBM

**Figure 1:**
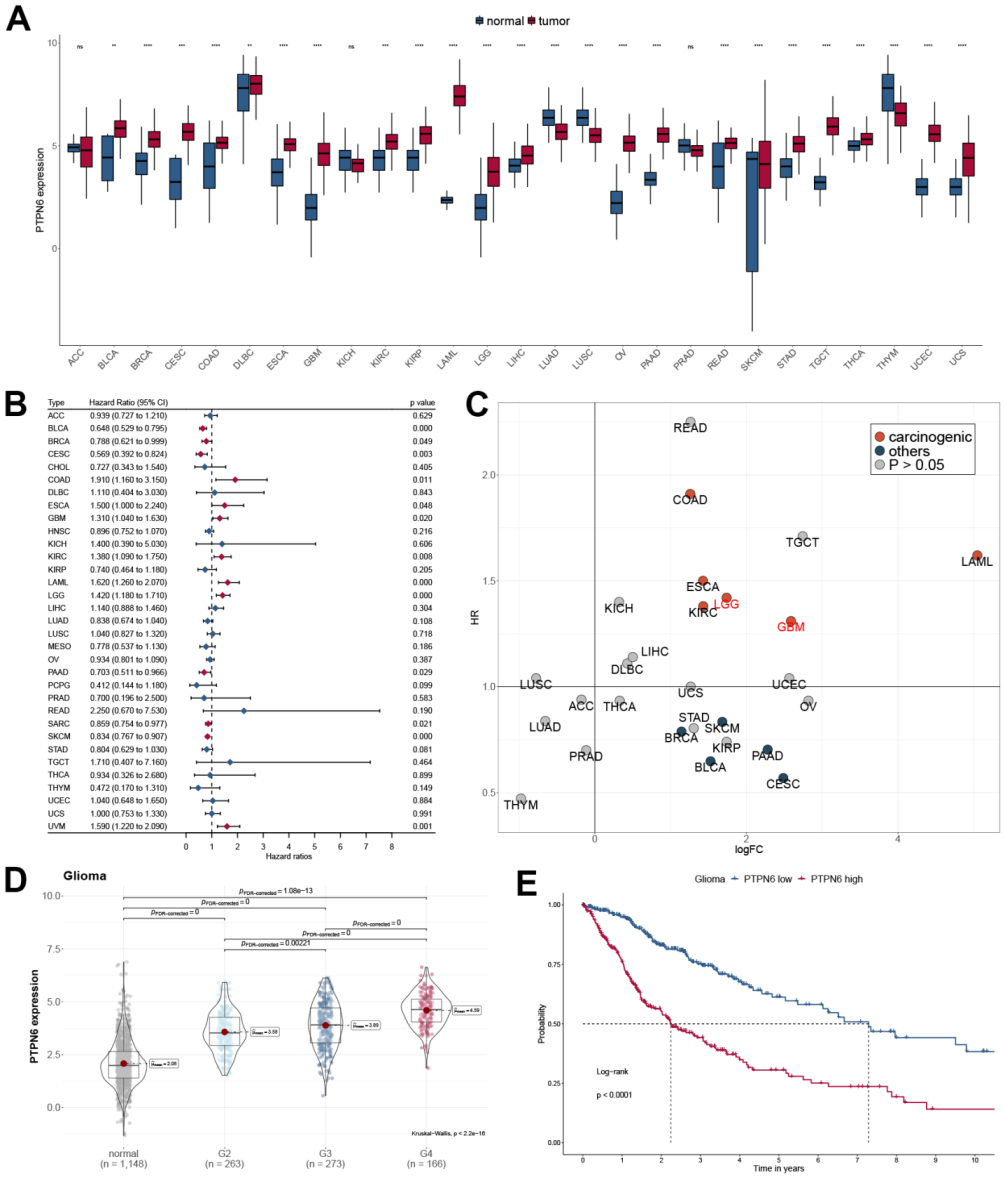
**Oncogenic properties of PTPN6 across pan-cancer.** (**A**) The gene expression of PTPN6 in cancer compared with normal tissues (**B**) Clinical significance of PTPN6 for overall survival in the TCGA dataset. (**C**) Underlying carcinogenesis of PTPN6 in cancer. (**D**) PTPN6 expression in different grades in GBM. (**E**) Survival analysis of PTPN6 expression levels for GBM patients.

We first investigated the PTPN6 gene expression in different cancer types using TCGA and GTEx databases. Interestingly, PTPN6 was significantly overexpressed in most cancer types, including GBM and LGG, while significantly downregulated in LUAD, LUSC, and THYM (Figure 1A). The overexpression of PTPN6 was also found in the other four independent glioma datasets (Supplementary Figure 1A). More importantly, the gene expression of PTPN6 was significantly related to different glioma subtypes in TCGA and CGGA datasets (Supplementary Figure 1B, 1C). We investigated the prognostic significance of PTPN6 in Pan-Cancer by applying the Cox regression model and log-rank test (Figure 1B and Supplementary Figure 2A–2C). Our analysis revealed a statistically significant correlation between PTPN6 overexpression and reduced survival in patients with GBM and LGG (Figure 1C). In addition, the high expression of PTPN6 was mainly related to advanced grade and poor OS in glioma (Figure 1D, 1E).

### Functional analysis of PTPN6 in pan-cancer and glioma

**Figure 2:**
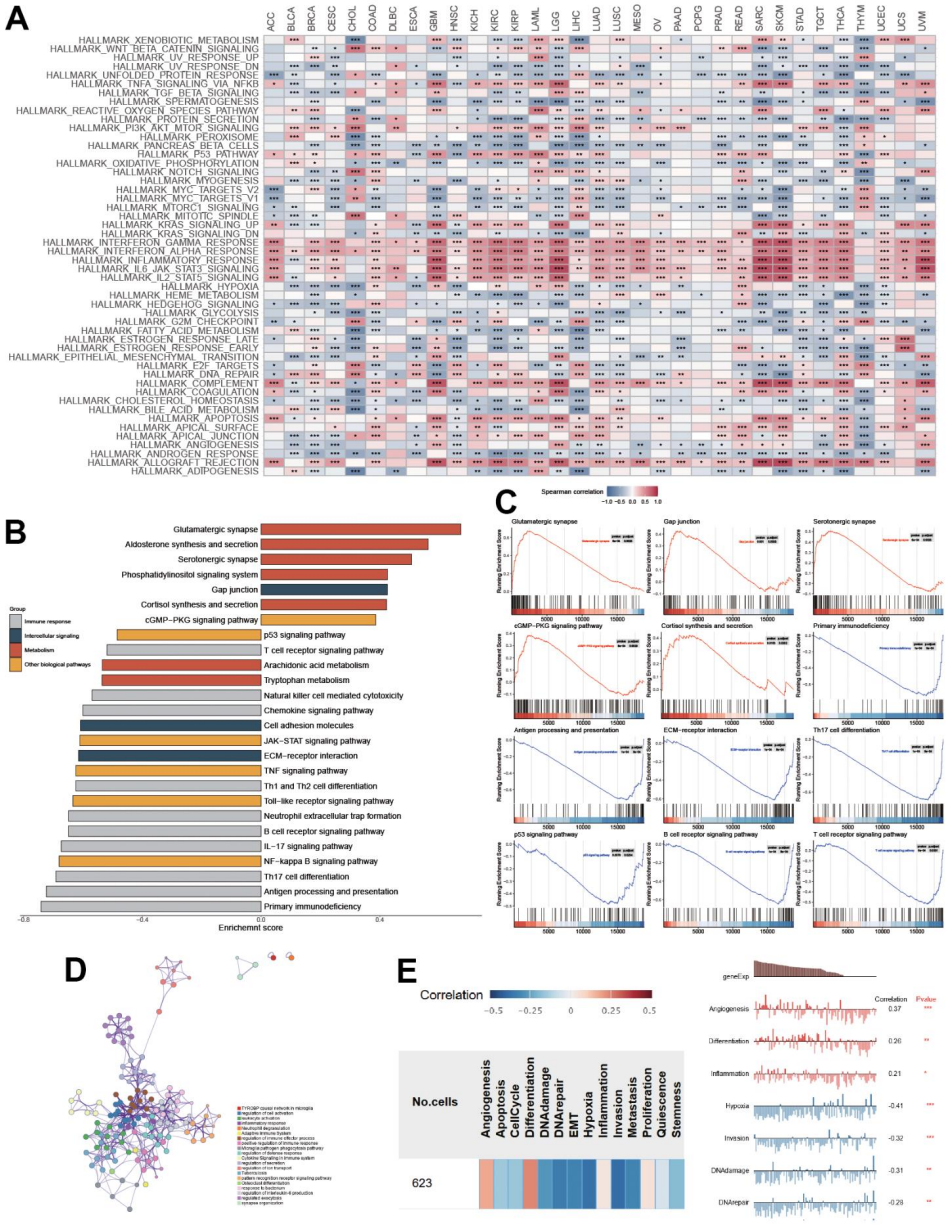
**Functional enrichment of PTPN6 in GBM.** (**A**) Correlation of PTPN6 with cancer hallmarks across different cancer. (**B**) Enriched pathways of PTPN6 expression in TCGA dataset. (**C**) Representational functions of PTPN6 in TCGA dataset. (**D**) Functional network of PTPN6 clustered by Metascape dataset. (**E**) Correlation of PTPN6 with angiogenesis, differentiation, inflammation, hypoxia, invasion, DNA damage and repair. *P < 0.05, **P < 0.01, ***P < 0.001.

To comprehensively explore the association between PTPN6 and cancer progression, we performed a Spearman correlation analysis between PTPN6 expression and cancer hallmark pathways in each cancer type (Figure 2A). Our functional analysis identified that several cancer hallmarks had been altered, including immune response, intercellular signaling, metabolism, and other biological factor pathways in LGG and GBM (Figure 2B). Additionally, several pathways, including gap junction, glutamatergic synapse, ErbB signaling pathway, serotonergic synapse, cGMP-PKG signaling pathway, and cortisol synthesis and secretion, were significantly up-regulated (Figure 2C, FDR < 0.05). Conversely, antigen processing and presentation, primary immunodeficiency, ECM receptor interaction, Th17 cell differentiation, p53 signaling pathway, and B/T cell receptor signaling pathways were significantly downregulated (Figure 2C, FDR < 0.05). We built a network of enriched GO terms and KEGG pathways of PNPN6 and its related genes based on the clusters and P-values by Metascape in GBM (Figure 2D). Notably, we found that the gene expression of PTPN6 was significantly and positively correlated with angiogenesis, differentiation, and inflammation, while it was negatively associated with hypoxia, invasion, DNA damage, and DNA repair in GBM (Figure 2E, P-value < 0.05).

### The green module involving PTPN6 was identified by WGCNA and functionally characterized in immune suppression

**Figure 3:**
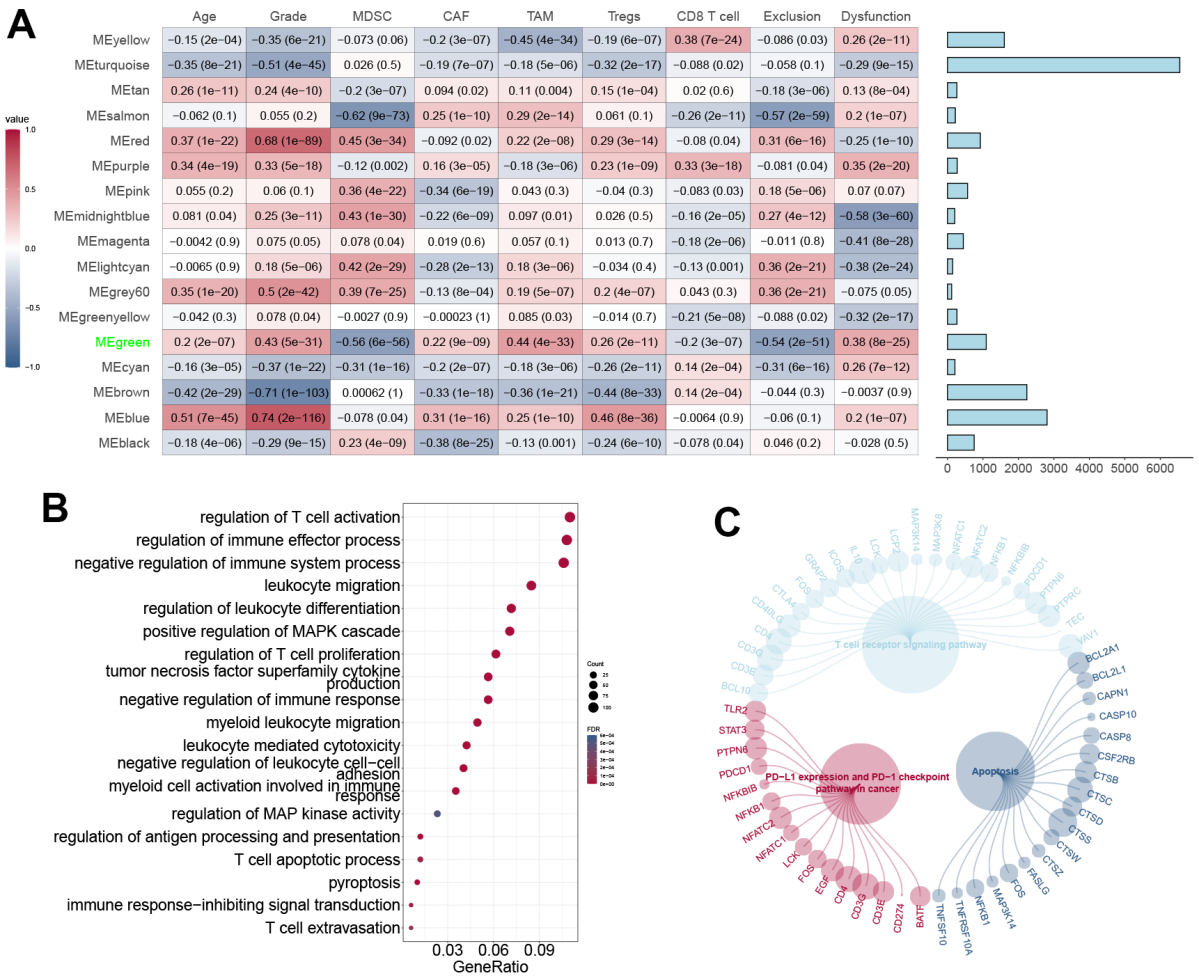
**WGCNA construction and module definition.** (**A**) Left: Module definition with immunosuppressive cells and CD8^+^ T cell dysfunction. Spearman correlation coefficient and P-value were indicated in the cells. Right: eigengenes in each block. (**B**) GO annotation of eigengenes in the MEgreen block. (**C**) Pathways of eigengenes in the MEgreen block.

CD8^+^ T cell infiltration is critical in predicting prognosis in GBM patients. We used CIBERSORT to assess CD8^+^ T cell levels and deleted outlier samples before running WGCNA. A power of 6 (scale-free R2 = 0.80) was found to be the soft threshold in our investigation (Supplementary Figure 3A). The data indicated that the dynamic tree-cut approach found 18 gene co-expression modules (Supplementary Figure 3B). Genes in the green module involving PTPN6 were strongly linked to GBM malignancy and poor prognosis by the heatmap of module trait correlations (Figure 3A), which indicated that genes in the green module might be responsible for GBM malignancy and prognosis. Module membership and gene importance were shown as scatter plots with similar results (Supplementary Figure 3C, 3D), which led to the green module being deemed the most important.

The biological processes analysis revealed that the green module’s genes were enriched by T cell activation, MAPK cascade, leukocyte migration, and proptosis (Figure 3B). The KEGG pathway analysis revealed the genes’ association with T-cell receptor signaling and apoptosis (Figure 3C). These data suggested that genes from the green module may mediate the TME in GBM.

### PTPN6 shapes the immunosuppressive TME in GBM

**Figure 4:**
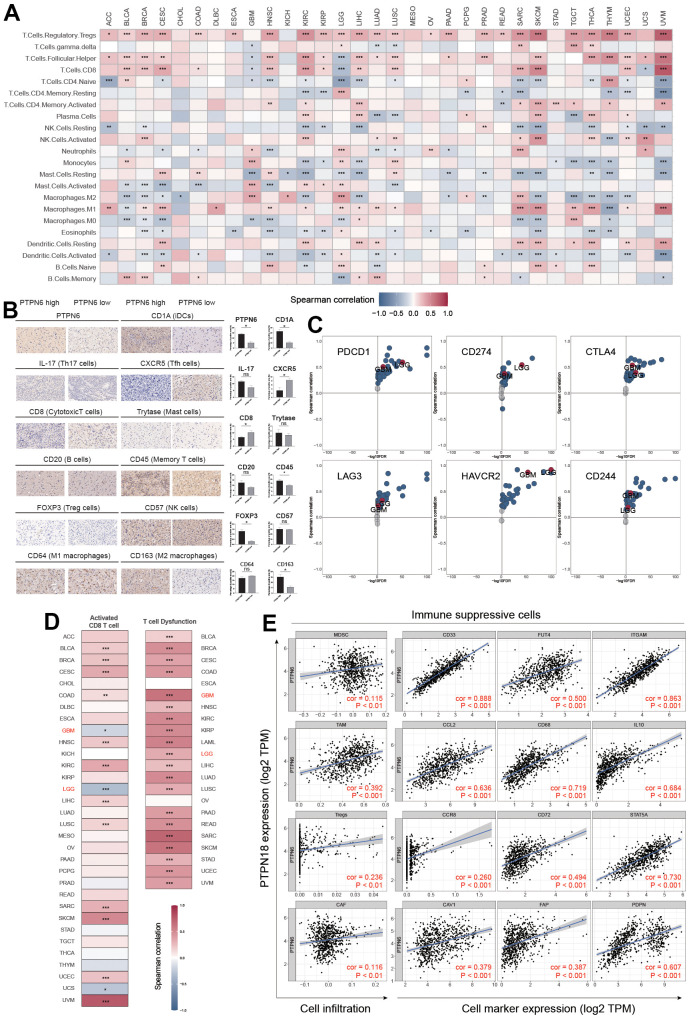
**PTPN6 shapes the immunosuppressive TME in GBM.** (**A**) Influence of PTPN6 with immune cell infiltration across different cancer. (**B**) TME comparison based on PTPN6 expression in GBM. (**C**) Association between PTPN6 and six immunosuppressive molecules across pan-cancer types. (**D**) Correlation of PTPN6 with activated CD8^+^ T cells and T cell exhaustion in different cancer types. (**E**) Correlation of PTPN6 with immunosuppressive cells and their representative marker genes. *P < 0.05, **P < 0.01, ***P < 0.001.

TME is an essential factor that affects tumor progression. To investigate the relationship between PTPN6 expression and infiltration levels of various immune cell types in GBM, we examined 22 immune cell types (Figure 4A). We validated the results by immunohistochemistry on GBM samples (Figure 4B). Surprisingly, we observed a positive correlation between PTPN6 expression and infiltration levels of M2 macrophages, regulatory T cells (Tregs), Th17 cells, and CD4+ memory T cells. At the same time, we found a negative correlation between PTPN6 expression and infiltration levels of B cells, mast cells, and CD8+ T cells.

Furthermore, we investigated the correlation between PTPN6 expression and the gene expression of immune checkpoint genes and immunosuppressive cells involved in CD8^+^ T cell exhaustion. We found that PTPN6 expression was positively correlated with PDCD1, CD274, CTLA4, LAG3, HAVCR2, and CD244 in most cancer types in TCGA, including LGG and GBM (Figure 4C). Additionally, we discovered a significant relationship between PTPN6 gene expressions and microsatellite instability (MSI) and tumor mutation burden (TMB) in several cancer types (Supplementary Figure 4), indicating that PTPN6 may have potential immunogenicity in these cancers. Moreover, we observed a negative correlation between PTPN6 expression and CD8^+^ T cell infiltration in LGG and GBM (Figure 4D).

We also investigated the role of immunosuppressive cells in the TME, including myeloid-derived suppressor cells (MDSC), tumor-associated macrophages (TAM), cancer-associated fibroblasts (CAF), and regulatory T cells (Tregs), which have been reported to inhibit CD8^+^ T cell infiltration and function. We found that PTPN6 expression was positively related to these four immunosuppressive cells and their corresponding marker genes (Figure 4E), suggesting that PTPN6 is correlated with immune suppression and CD8^+^ T cell exhaustion in GBM.

### Single-cell sequencing of PTPN6 expression on GBM

**Figure 5:**
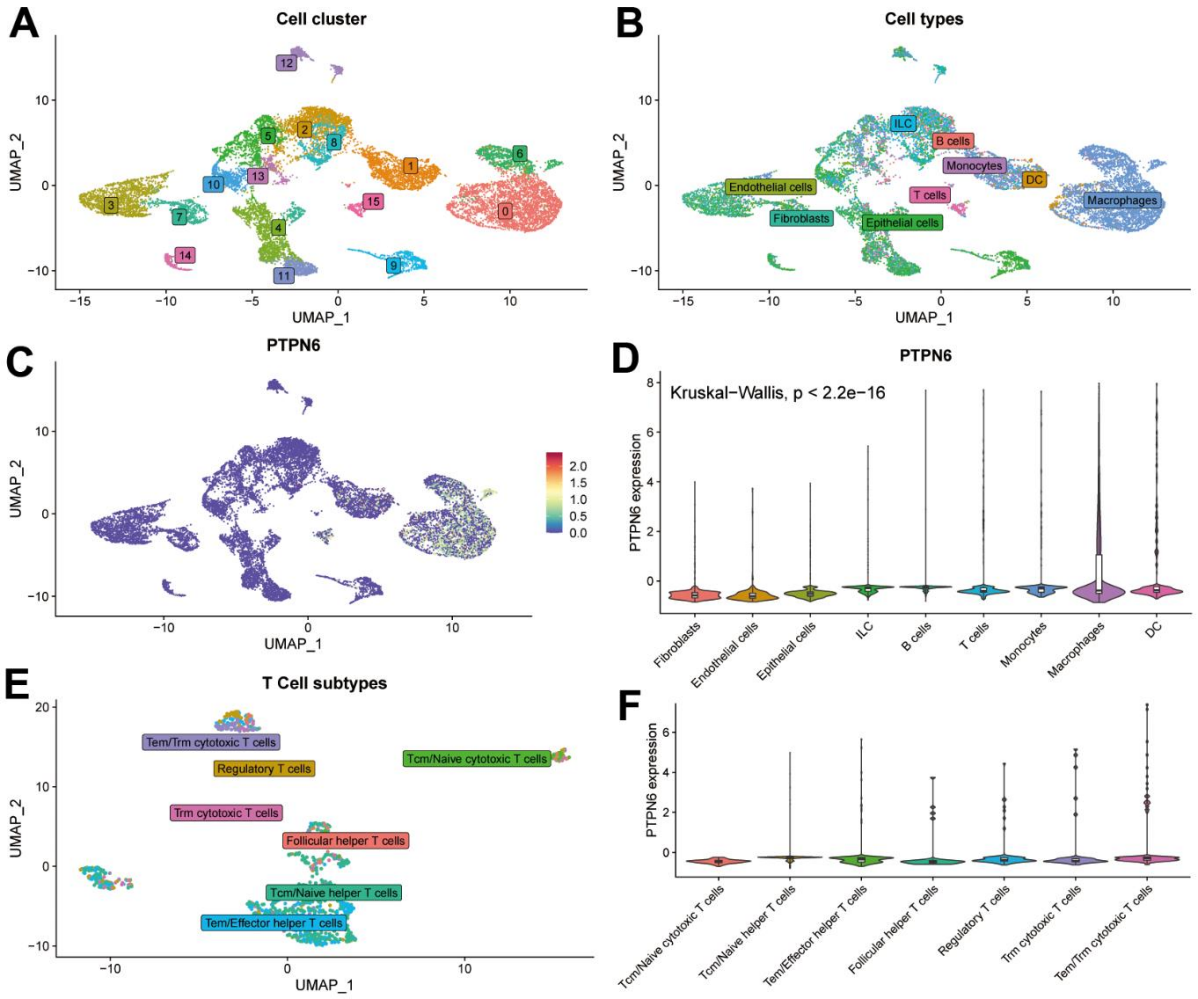
**Single-cell sequence analyzing PTPN6-related cell type distribution in GBM.** (**A**) Cell clusters of single-cell sequence in GSE131928. (**B**) Cell annotation of single-cell sequence in GSE131928. (**C**) UMAP plot showing expression and distribution of PTPN6 in GSE131928 database. (**D**) Barplot showing expression of PTPN6 in different cell. (**E**) Cell annotation of single-cell sequence in the subtype of T cells. (**F**) Barplot showing expression of PTPN6 in different subtype of T cells.

To confirm the expression of PTPN6 and its significance in the TME of GBM, we applied a single cell profile of 28 GBM patients containing 24,131 single cells to analyze the correlation. The cell clusters were shown in Figure 5A. The findings showed that PTPN6 was predominately concentrated in DC and macrophages cells, whereas T cells and B cells exhibited lower expression, which accords with the bulk RNA-seq data from the TCGA dataset (Figure 5B–5D). Further analysis showed that PTPN6 was the comparatively low expression in every subtype of T cells (Figure 5E, 5F). These findings indicate that the expression level of PTPN6 was significantly variable in various immune cell types, which may be the root of GBM microenvironmental heterogeneity and was related to GBM tumor progression.

### PTPN6 promoted cancer progression in GBM

**Figure 6:**
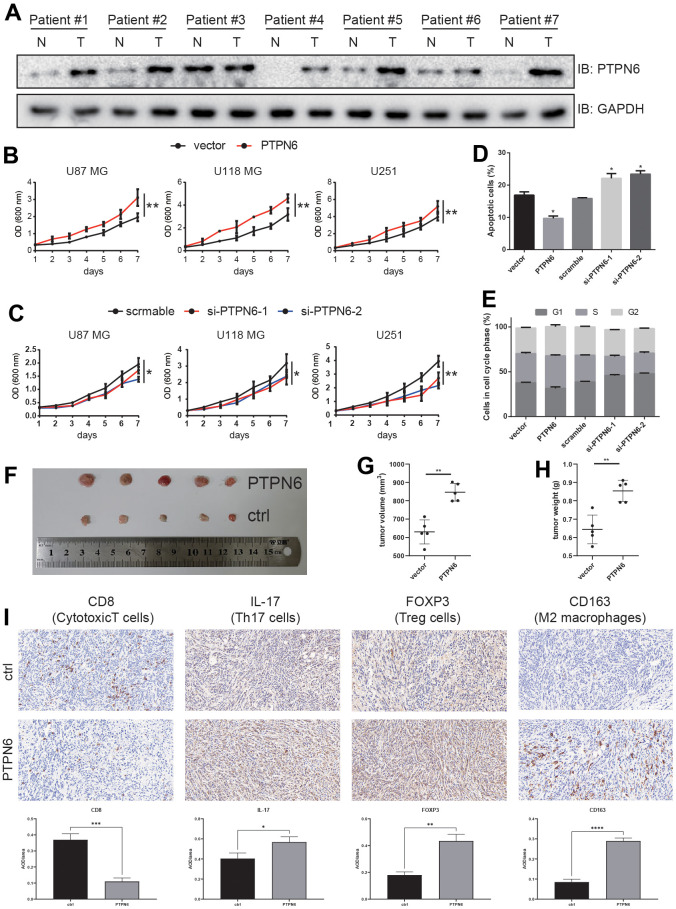
**PTPN6 promotes GBM progression.** (**A**) PTPN6 protein expression level is up-regulated in GBM human samples compared with the paired adjacent tissue. (**B**) PTPN6 overexpression accelerates glioma cell proliferation. (**C**) PTPN6 knockdown inhibits glioma cell proliferation. (**D**) PTPN6 overexpression inhibits glioma cell apoptosis; and promotes cell apoptosis after PTPN6 knockdown. (**E**) PTPN6 overexpression promotes cell cycles; and inhibits cell cycles after PTPN6 knockdown. (**F**) PTPN6 promotes GBM tumorigenesis *in vivo*. (**G**, **H**) Calculation of GBM nidus from (**F**), **P < 0.01. (**I**) Evaluating the GBM TME in C57 mice (n = 6, biological repetition = 3). *P < 0.05, **P < 0.01, ***P < 0.001, ****P < 0.001.

To investigate whether PTPN6 was associated with GBM tumorigenesis, we analyzed the genomic alterations of PTPN6. Our analysis revealed that PTPN6 exhibited a relatively low frequency of mutations but a high frequency of copy number variations (CNVs) in GBM (Supplementary Figure 5). Moreover, we confirmed our findings by examining clinical specimens, which showed that PTPN6 was significantly overexpressed in GBM samples compared to paired adjacent samples at the protein level (Figure 6A). To explore the effect of PTPN6 on cell growth, we performed ectopic expression and knockdown experiments. Our results indicated that PTPN6 overexpression promoted growth velocity (Figure 6B and Supplementary Figure 6A) while PTPN6 knockdown suppressed cell growth (Figure 6C and Supplementary Figure 6B) in different cell lines of GBM. Additionally, we observed that PTPN6 could inhibit cell apoptosis and promote tumor proliferation in glioma cell lines (Figure 6D, 6E and Supplementary Figure 6C, 6D).

To further elucidate the role of PTPN6 in glioblastoma development, we investigated immune cell infiltration in C57BL/6 mice. Our results demonstrated that mouse glioblastoma cells stably expressing PTPN6 significantly increased tumor development (Figure 6F–6H). Furthermore, we found that PTPN6 blocked the penetration of CD8^+^ T cells into the tumor lesion while promoting the infiltration of Th17 cells and immunosuppressive cells, such as Tregs and M2 macrophages, into tumors (Figure 6I).

### Integrative analysis of PTPN6 on immunotherapy and drug response

**Figure 7:**
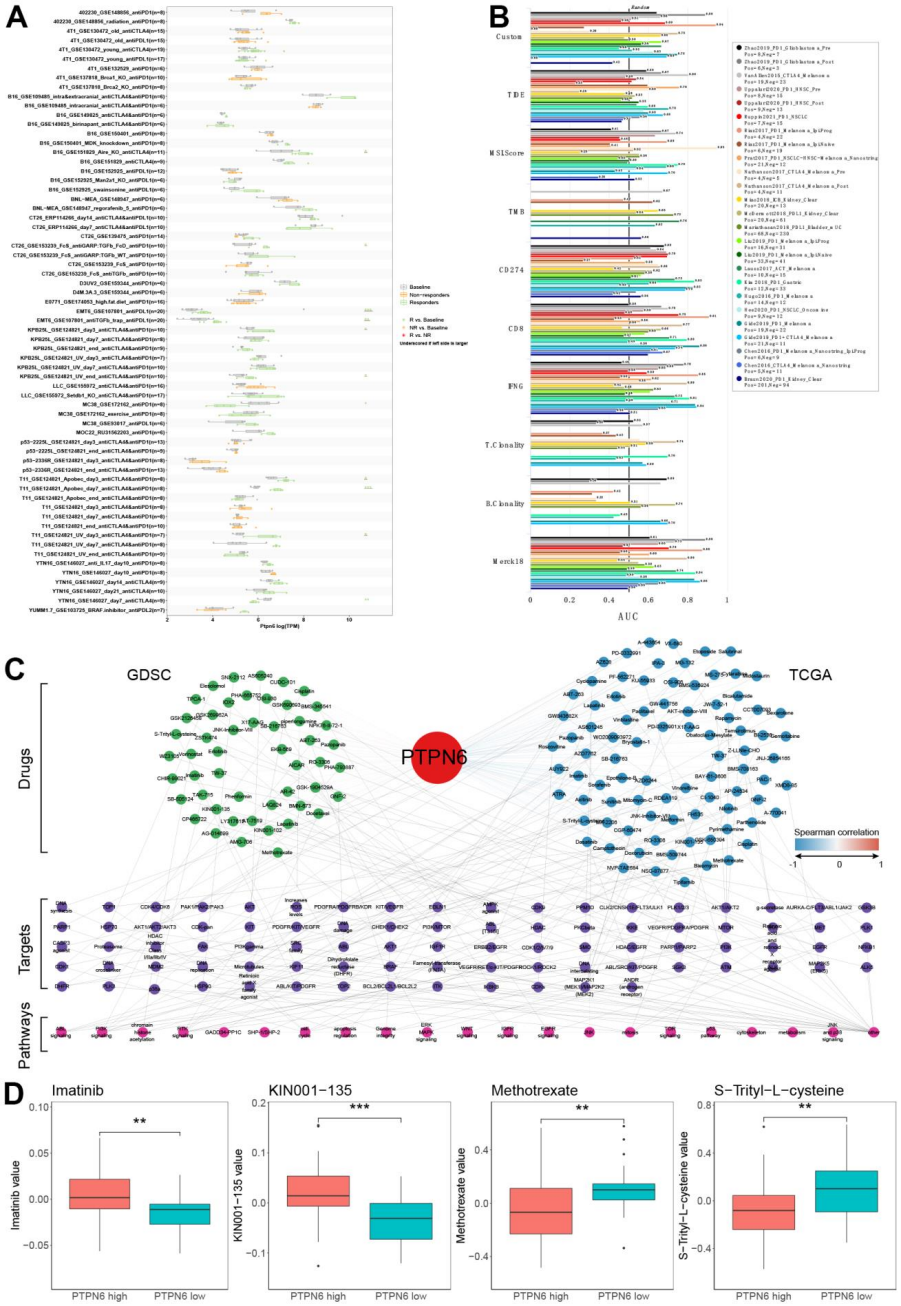
**Evaluation of PTPN6 clinical significance.** (**A**) Immunotherapy response of PTPN6 in immunotherapy cohorts. (**B**) Biomarker relevance of PTPN6 in immunotherapy cohorts. (**C**) Anti-drug response of PTPN6 in GDSC and TCGA databases. (**D**) Representative drugs in TCGA database.

To investigate the potential utility of PTPN6 as a novel immune target in pan-cancer, we analyzed the association between PTPN6 expression and immunotherapy response as well as drug sensitivity (Figure 7). Importantly, we found that higher expression levels of PTPN6 were associated with increased immunotherapy response in 9 different immunotherapy groups (Figure 7A). Additionally, we compared PTPN6 to other established biomarkers based on their ability to predict immunotherapy response in human immunotherapy cohorts. Notably, we found that PTPN6 had an AUC value above 0.5 in 15 out of 25 immunotherapy cohorts, outperforming other biomarkers such as MSI score, TMB, T. Clonality, and B. Clonality (Figure 7B). CD247, TIDE, IFNG, and CD8 had better predictive values than PTPN6, with AUC values above 0.5 in 21, 18, 17, and 18 immunotherapy cohorts, respectively.

To assess the potential responsiveness of PTPN6 to anti-cancer drugs, we evaluated the correlation between PTPN6 expression and drug sensitivity for 252 anti-cancer drugs using the GDSC dataset across 1,074 cancer cell lines. We also conducted an integrative analysis of PTPN6 and response to anti-cancer therapies in TCGA patients. Our combined analysis of GDSC and TCGA data revealed that PTPN6 significantly correlated with 100 drugs in each database (Figure 7C). Four drugs showed similar effects in both databases: Imatinib, KIN001-135, Methotrexate, and S-Trityl-L-cysteine (Figure 7D). These results provide important insights into the potential mechanisms underlying PTPN6’s effects on immune intervention and patient survival.

## DISCUSSION

PTPN6 participates in various pathway regulations and is conceivable as a drug target in some types of cancer [15]. While PTPN6 has been extensively studied in other cancer types, such as bladder cancer [16] and colorectal cancer [17], its roles in GBM have not been thoroughly explored. In this study, we identified that PTPN6 was significantly overexpressed in GBM patients in TCGA, which was further validated using other independent datasets and human specimens at the protein level. PTPN6 overexpression was significantly associated with poor survival and advanced grade in GBM, suggesting its oncogenic properties [16]. Our observations were in line with previous research that demonstrated the association of PTPN6 with poor prognosis in patients with bladder cancer and myelodysplastic syndromes [11] and its potential role as a tumor suppressor in esophagus cancer [18]. Our results indicated that PTPN6 could be a prognostic marker for patients with GBM.

PTPN6 expression was positively associated with the TME formation of TAMs, Tregs, Th17 cells, and CD4^+^ memory T cells while reversely associated with those of B cells, mast cells, and CD8^+^ T cells. Further correlation analysis revealed that PTPN6 expression was positively related to the expression of six immune checkpoint genes (PDCD1, CD274, CTLA4, LAG3, HAVCR2, and CD244) while negatively correlated with CD8^+^ T cell infiltration in GBM. Additionally, PTPN6 expression was positively related to the four immunosuppressive cells, including myeloid-derived suppressor cells, tumor-associated macrophage, Tregs, and cancer-associated fibroblasts. Other studies have shown that targeting PTPN6 may be an attractive therapeutic method for increasing the ability of leukocytes to fight cancer [14]. Furthermore, in zebrafish embryos, the lack of the PTPN6 gene results in an overactivated innate immune system and impaired ability to fight off bacterial infections [19], suggesting its role in inhibiting immune responses and enhancing tumor progression in GBM.

GO and KEGG enrichment analyses demonstrated that PTPN6 was significantly and positively correlated with angiogenesis, differentiation, and inflammation, while negatively associated with hypoxia, invasion, DNA damage, and DNA repair. Recent studies have shown that PTPN6 inhibits Caspase-8 and Ripk3/Mlkl-dependent inflammation and regulates HIF-1α protein levels in endothelial cells under hypoxia [17, 20]. Our functional experiments further confirmed the oncogenic properties of PTPN6 in GBM, as PTPN6 overexpression promoted tumor growth and colony formation in cell lines and nude mice.

Our study found that PTPN6 was an effective prognostic marker for patients with GBM. Moreover, we discovered that PTPN6 regulated tumor progression by reducing immune cell infiltration and inhibiting immune response, indicating that PTPN6 could be a therapeutic target for cancer immunotherapy in GBM.

## MATERIALS AND METHODS

### Data resource

Gene expression profiles, somatic mutations, copy number variations (CNV), and clinical data of pan-cancer (Level 3) were extracted using the TCGAbiolinks package in R [21]. MC3 project containing somatic variants information was obtained from the previous research [22]. Copy number variations (CNV) were retrieved from Broad GDAC Firehose and preprocessed using GISTIC2 [23].

The clinical information was downloaded from the reported article [14] or through the TCGAbiolinks R package. Genotype tissue profiles were downloaded using the UCSC Xena project. Other glioma data were downloaded from the CGGA (CGGA.mRNAseq_693) and GlioVis (Rembrandt, Gravendeel, and Gill) databases [24, 25]. The single-cell data of GBM were acquired from the GEO database (GSE131928) [26]. Detailed information of cohorts used in this study was list in Supplementary Table 1.

### Survival analysis

Kaplan-Meier methods performed in the R package assessed overall survival (OS) between the target groups. The log-rank test was leveraged to evaluate the statistical data; a P-value < 0.05 was considered significantly different.

### WGCNA analysis

The present study employed WGCNA to conduct a correlation analysis between PTPN6 expression and clinical as well as immunological parameters. The resulting correlation matrix was subjected to a predetermined power and clustered via the WGCNA package in R, using the following parameters: power = 20, TOMType = “signed”, pamStage = F, and minModuleSize = 3, as we described before [27, 28].

### Functional analysis

Gene set enrichment analysis (GSEA) was leveraged via GSEA software with gene sets from the Molecular Signatures Database [29–31]. FDR corrected q-value < 0.05 was regarded to be significant statistically. The Metascape was used to build the interactive network of PTPN6 and its related genes. The analysis of biological progresses for PTPN6 was decoded using CancerSEA. Further study was conducted on the significant results where the p-value was less than 0.05 [32].

### TME evaluation

The influence of PTPN6 on TME formation was assessed using a previous study as a reference [33]. TIDE was used to determine the response to cancer immunotherapy based on the expression of PTPN6 and the T cell exclusion and dysfunction [34].

### Drug response analysis

Gene expressing matrix and area under the dosage response curve of glioma cell lines were collected from the GDSC database [35, 36]. Correlation coefficient |Rs| > 0.25 and FDR < 0.05 were employed to further analysis [37]. In addition, previously conducted studies were used to obtain imputed tumor response data for 138 anti-cancer drugs, which were subsequently used to assess treatment response for GBM patients [38].

### Cell lines and human tissues

The U87, U118-MG, and U-251-MG glioma cell lines were cultured in DMEM supplemented with 1% Penicillin/Streptomycin and 10% fetal bovine serum and maintained at 37° C and 5% CO2. Human tissue samples were obtained from patients undergoing clinical surgery at the Second Affiliated Hospital of Bengbu Medical College, and clinical information were list in Supplementary Table 2.

### Stable cell line construction

We established a stable overexpression PTPN6 cell line in human glioblastoma cells using lentiviral-based gene transfer. The full-length PTPN6 cDNA was cloned into a lentiviral expression vector, and viral particles carrying the PTPN6 gene were generated and used to transduce the glioblastoma cells. Positively transduced cells were selected and expanded to create a stable pool of overexpression PTPN6 cells. The successful overexpression of PTPN6 was validated using quantitative PCR and Western blot analysis. Functional experiments were conducted to assess the impact of PTPN6 overexpression on cell proliferation and tumorigenesis in mice. These experiments aimed to investigate the role of PTPN6 in glioblastoma cell behavior and its potential as an immunotherapeutic target.

### Transfection and Western blotting

Transient transfection of plasmids and siRNAs was applied using Lipofectamine 3000 (Thermo Fisher, USA) according to the manufacturer’s instructions. After 48 hours, total proteins were extracted from the cultured cells and quantified using a normal quantitative system. Western blotting was conducted using standard techniques, with GAPDH antibody as an internal control for normalization. The blots were stripped and probed with primary antibodies against PTPN6 (#3759) and GAPDH (#5174) from CST company. The imaging system were utilized for blot scanning, visualization, and analysis. All experiments were repeated thrice with consistent outcomes.

### Immunohistochemistry

The GBM tumor tissue was fixed with 4% paraformaldehyde, embedded in paraffin, and subsequently cut into 4 μm sections. The paraffin sections were incubated overnight at 4° C with antibodies according to standard protocols. Two independent, blinded pathologists independently evaluated each section. Two independent blinded pathologists assessed each section separately. Immunohistochemistry was performed using antibodies against PTPN6 (ab32559, Abcam), CD1A (17325-1-AP, Proteintech), IL-17 (ab79056, Abcam), CXCR5 (ab254415, Abcam), CD8 (66868-1-Ig, Proteintech), Tryptase (ab2378, Abcam), CD20 (10252-1-AP, Proteintech), CD45 (60287-1-Ig, Proteintech), FOXP3 (ab20034, Abcam), CD57 (19401-1-AP, Proteintech), CD64 (ab140779, Abcam) and CD163 (ab79056, Abcam).

### Cell cycle assay

Glioma cell lines treated with transfection of target oligos were washed twice with PBS that had been pre-chilled in preparation for the cell cycle assay. Subsequently, the cells were fixed overnight at 4° C with pre-cooled 70% ethanol. After washing once with 1 mL of PBS, the cells were treated with a PBS solution containing 50 μg/mL propidium iodide, 100 μg/mL RNase A, and 0.2% Triton X-100. 30 minutes were spent incubating the cells at 4° C and in the dark. 20,000 cells were enumerated using a BD flow cytometer after standard cell cycle procedures. ModFit software was used to analyze the results.

### Cell apoptosis assay

The cells were rinsed once with PBS buffer, and FITC-Annexin V and PI at a final concentration of 1 μg/mL were added to the PBS buffer. 10-15 minutes were spent incubating the mixture in the dark at room temperature. At 488 nm, the flow cytometer measured the fluorescence, gathering FITC and PI-PE signals. Flowjo software was used to analyze the results.

### MTT assay

Cells were seeded in 96-well plates at a density of 1.2 × 10^5^ cells per well and incubated at 37° C for 4 hours in the presence of 5 mg/mL MTT (Sigma-Aldrich) dissolved in PBS. After removal of the supernatant, the formazan crystals were dissolved in 100 μL of DMSO, and the optical density was measured at 570 nm using a microplate reader (Bio-Tek). The IC50 values for two cell lines were calculated based on the cytotoxicity obtained from the MTT assay. Each experiment was repeated at least three times.

### Tumorigenesis in mice

C57BL/6 mice were obtained from Charles River (Beijing, China) and maintained in a pathogen-free environment. The Ethics Committee of Bengbu medical college approved all animal experiments. Six mice per group were subcutaneously injected with either murine glioma GL261 expressing PTPN6 stably or empty vector (6 × 10^7^ cells in serum-free DMEM) in the right super lateral tissue. After 10 days, mice were sacrificed, and the protein expression of the target gene was evaluated by Western blot analysis.

### Statistical analysis

The data were analyzed using R software (version 4.0.0; https://cran.r-project.org/). To compare variables with normal and non-normal distributions, the student’s t-test and Wilcoxon rank-sum test were employed, respectively. To control the false discovery rate (FDR), two-sided P-values were corrected using the Benjamin-Hochberg (BH) method. A P-value less than 0.05, after adjusting for BH effects, was considered statistically significant unless otherwise specified.

## Supplementary Material

Supplementary Figures

Supplementary Tables
