# Supplementary material for: Single-cell and bulk sequencing analyses reveal the immune suppressive role of PTPN6 in glioblastoma
Source: Aging (Albany NY). 2023 Sep 21;15(18):9822–41. doi: 10.18632/aging.205052 (PMC10564408; doi:10.18632/aging.205052)
Supplement: Supplementary Figures [file aging-15-205052-s001.pdf]

SUPPLEMENTARY FIGURES

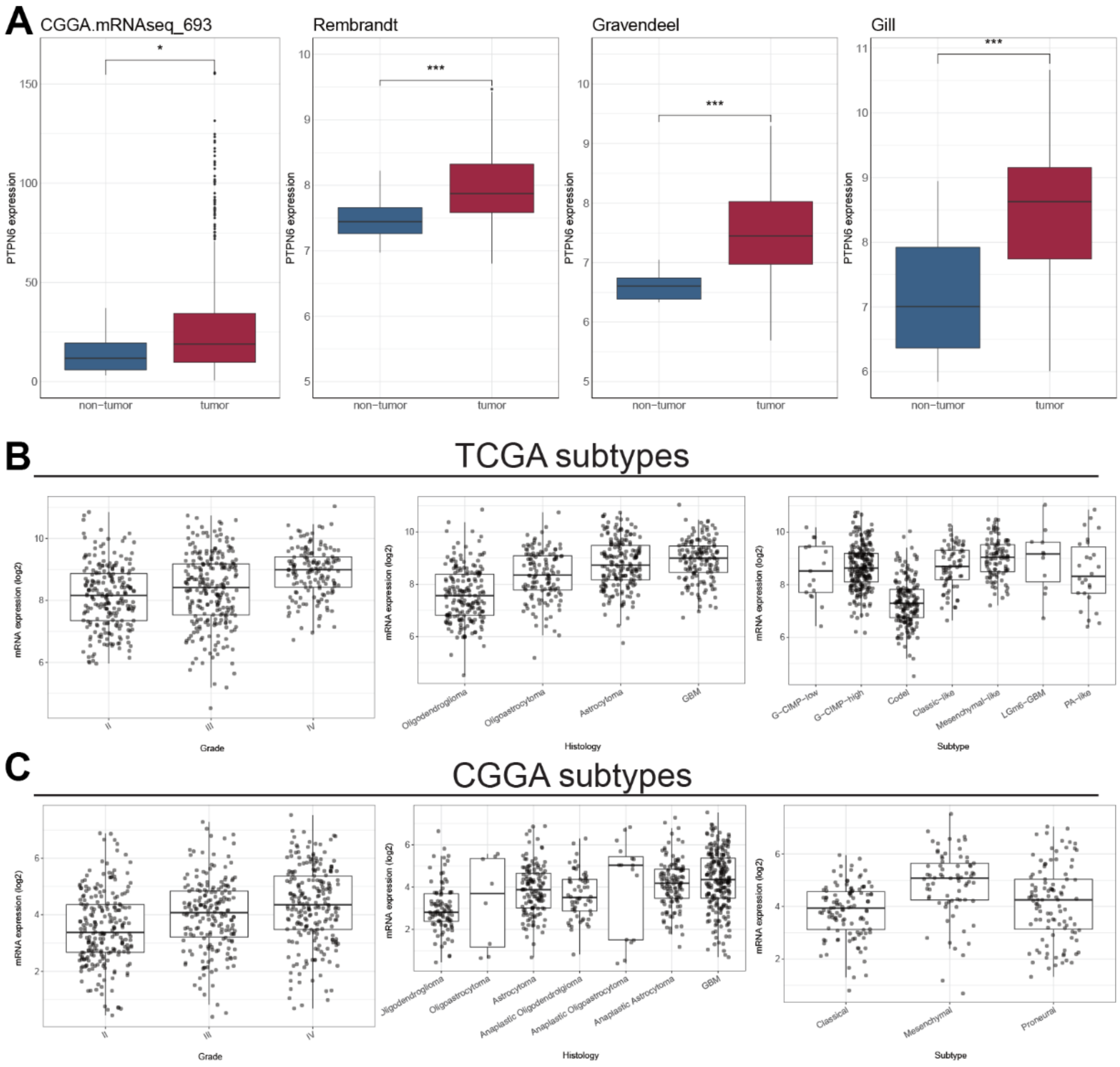

Supplementary Figure 1. Expression pattern of PTPN6 in GBM. (**A**) Distribution of PTPN6 expression from four different datasets. (**B**) Distribution of PTPN6 expression in TCGA subtypes. (**C**) Distribution of PTPN6 expression in CGGA subtypes.

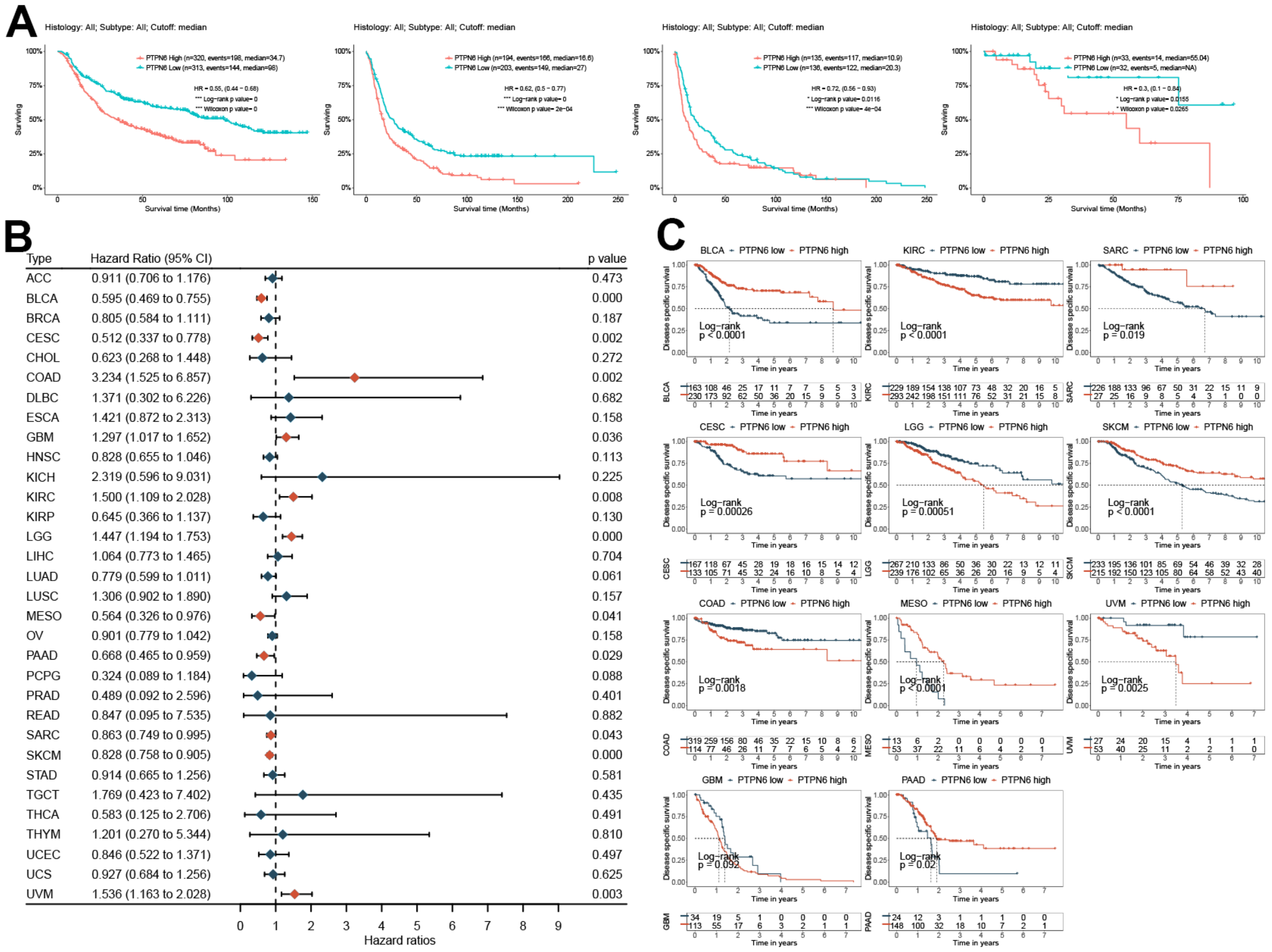

Supplementary Figure 2. Prognostic analysis of PTPN6 for disease-specific survival in pan-cancers. (**A**) Clinical prognosis of PTPN6 in four different datasets. (**B**) Clinical significance of PTPN6 for disease specific survival in the TCGA dataset. (**C**) Representative survival analysis of PTPN6 in TCGA datasets.

0

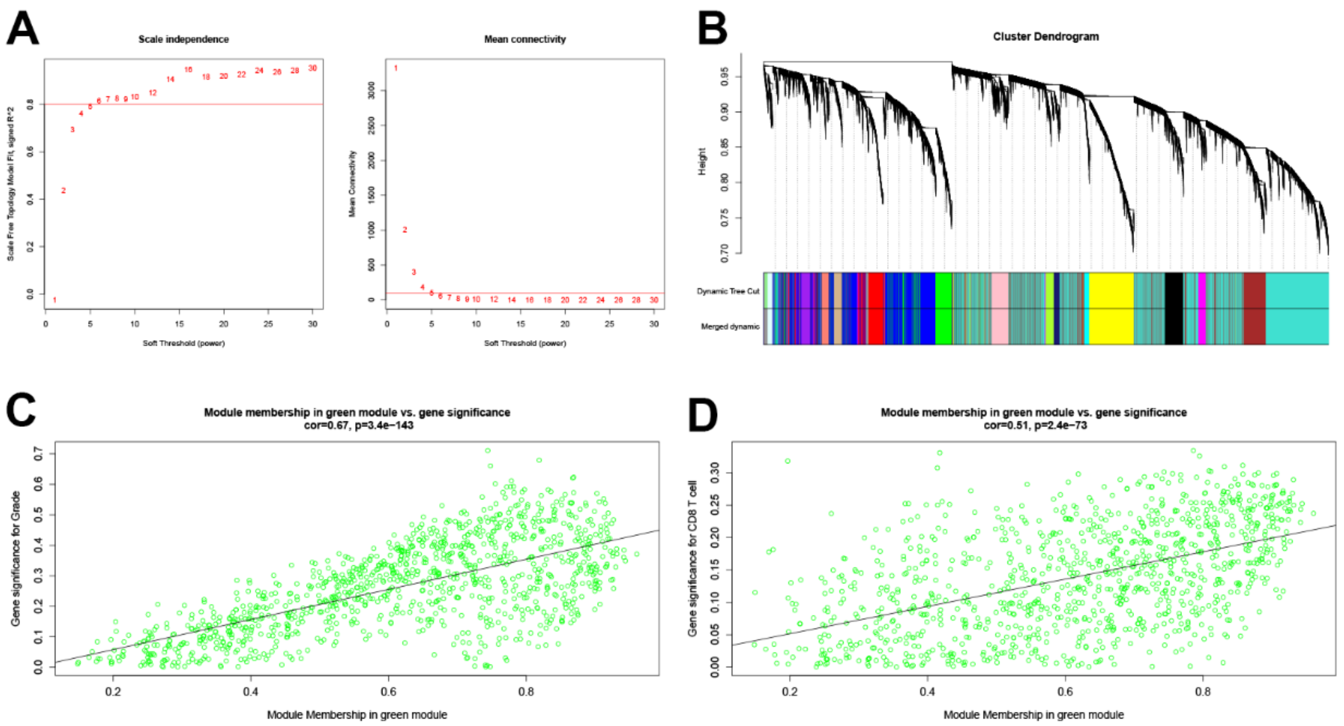

Supplementary Figure 3. WGCNA construction and functional annotation. (**A**) Power distribution of WGCNA. (**B**) Dynamic tree of 18 modules. (**C**, **D**) Module membership and gene importance.

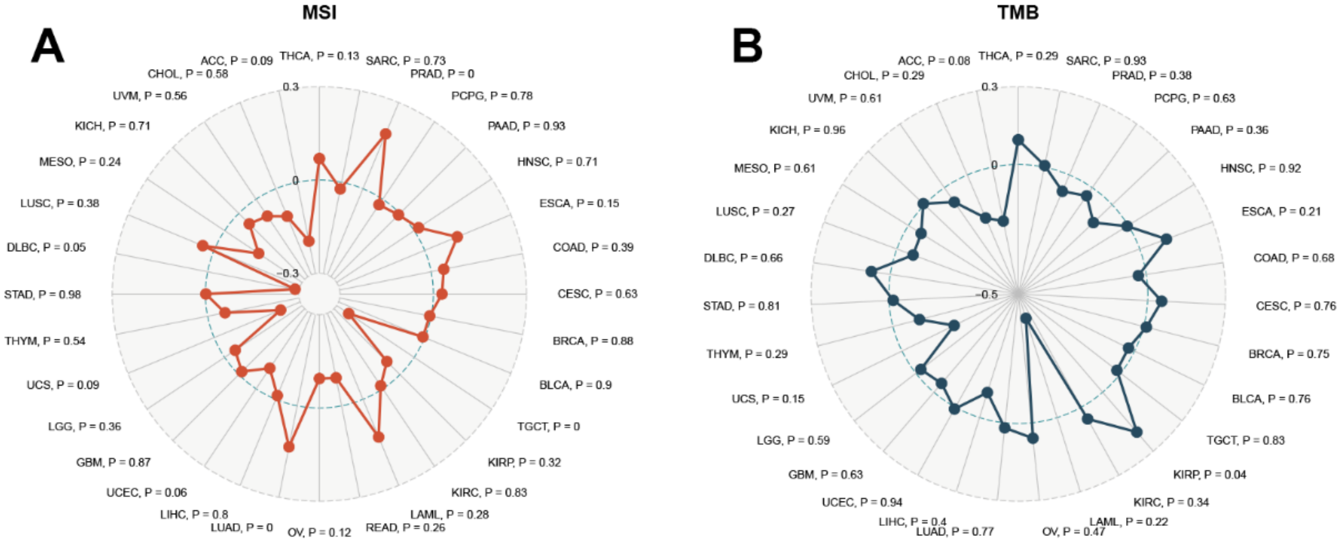

Supplementary Figure 4. Correlations between PTPN6 and TMB, MSI in pan-cancers. (**A**) MSI of PTPN6 for TCGA datasets. (**B**) TMB of PTPN6 for TCGA datasets.

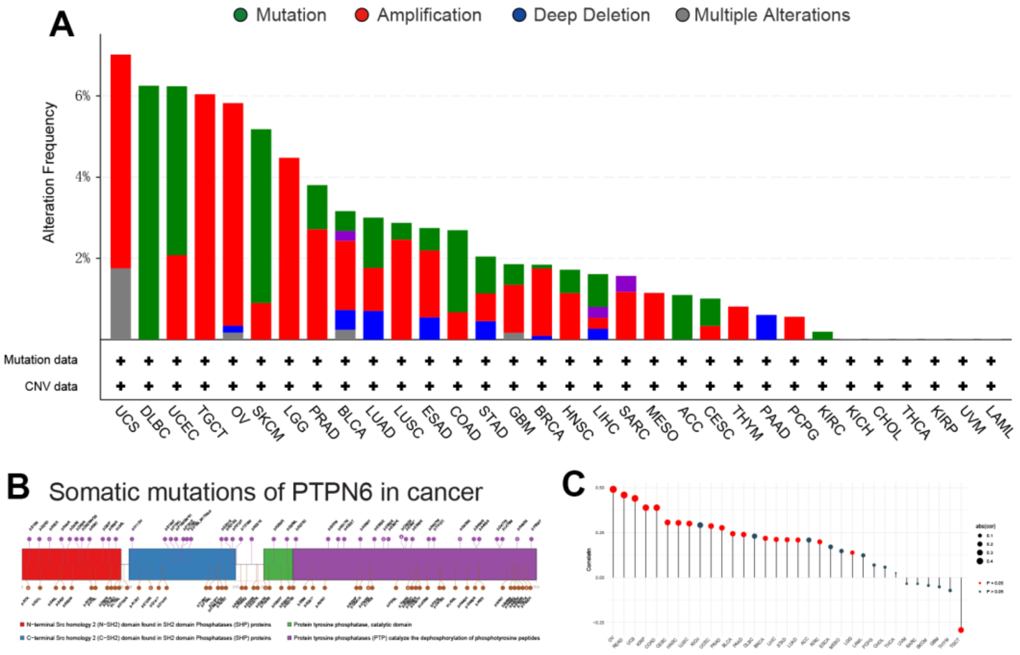

Supplementary Figure 5. Genetic alterations of PTPN6 in pan-cancers. (**A**) Mutational frequency of PTPN6 in TCGA datasets. (**B**) Somatic mutations of PTPN6 in cancer. (**C**) CNV of PTPN6.

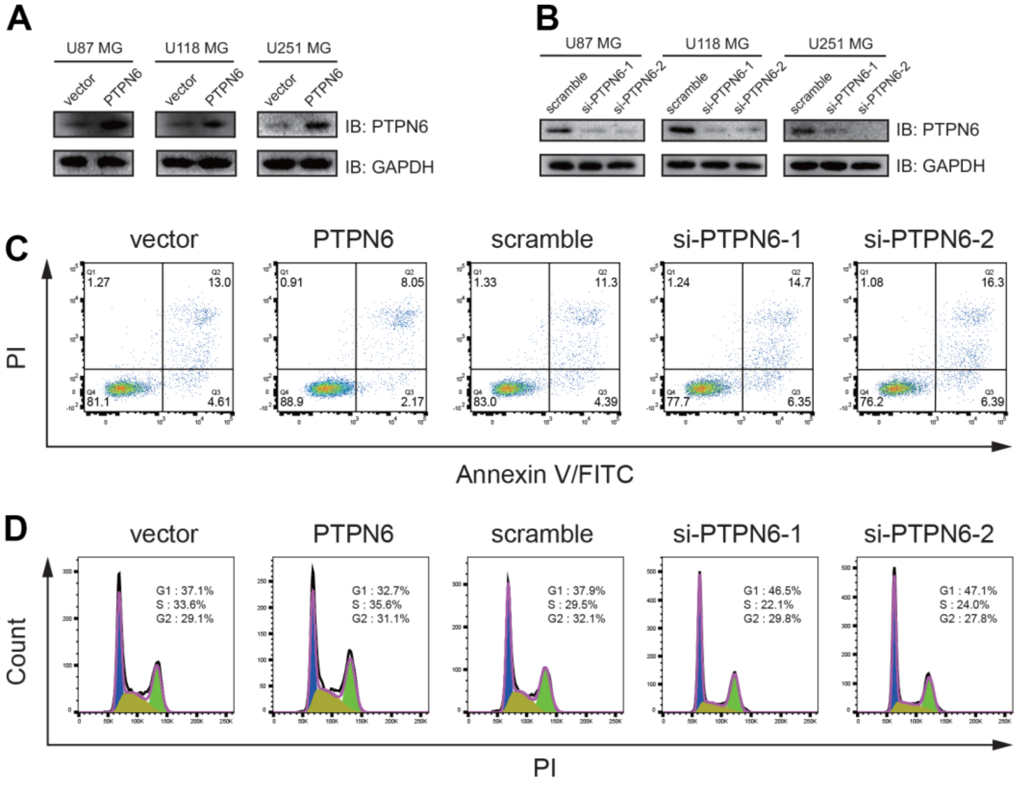

Supplementary Figure 6. PTPN6 regulated cell cycle and apoptosis in glioma cells. (**A**) PTPN6 was transduced in glioma cell lines. (**B**) PTPN6 was knockdown in glioma cell lines. (**C**) Flow cytometric analysis of early and late apoptotic cells with annexin V and propidium iodide (PI). (**D**) The effect of PTPN6 on cell cycle distribution.
