# Supplementary material for: Single-cell and bulk sequencing analyses reveal the immune suppressive role of PTPN6 in glioblastoma
Source: Aging (Albany NY). 2023 Sep 21;15(18):9822–41. doi: 10.18632/aging.205052 (PMC10564408; doi:10.18632/aging.205052)
Supplement: Supplementary Tables [file aging-15-205052-s002.pdf]

SUPPLEMENTARY TABLES

Supplementary Table 1. Detailed information of included clinical cohorts.

Cohort name	Data source	Sample number	Survival data	Platform
GTEx	GTEx	8295	No	RNA-seq
TCGA	TCGA	9807	Yes	RNA-seq
CGGA_mRNAseq_693	CGGA	693	Yes	RNA-seq
Rembrandt	GlioVis	580	Yes	Microarray
Gravendeel	GlioVis	284	Yes	Microarray
Gill	GlioVis	92	No	RNA-seq
GSE131928	GEO	24131	No	RNA-seq

Supplementary Table 2. Clinical characteristics of GBM patients (n = 30).

Variable	No	%
Age		
< = 55	8	26.7
> 55	22	73.3
Gender		
Female	19	63.3
Male	21	36.6
Grade		
II	2	6.67
III	13	43.33
IV	15	50.0
